# Supplementary material for: Topotactically transformable antiphase boundaries with enhanced ionic conductivity
Source: Nat Commun. 2023 Nov 15;14:7382. doi: 10.1038/s41467-023-43086-5 (PMC10651924; doi:10.1038/s41467-023-43086-5)
Supplement: Supplementary file 1 — Supplementary Information [file 41467_2023_43086_MOESM1_ESM.pdf]

Supplementary Information

Topotactically transformable antiphase boundaries with enhanced ionic conductivity

Kun Xu et al.

This file includes:

Supplementary Figure 1. Structural characterization of as-grown $Sr_4Fe_6O_{13-\delta}$ film by X-ray diffraction and the measurement of the conductivity.

Supplementary Figure 2. Low-magnified scanning transmission electron microscopy-high angle annular dark field image of as-grown $Sr_4Fe_6O_{13-\delta}$ film.

Supplementary Figure 3. Atomic-scale composition analysis of non-topotactically transformable APBs.

Supplementary Figure 4. Atomic-scale composition analysis of topotactically transformable APB-IV.

Supplementary Figure 5. The quantitative analysis of the ratios of integrated intensities of peaks A and B.

Supplementary Figure 6. Atomic-scale composition and structure analysis of topotactically transformable APB-III.

Supplementary Figure 7. Schematic illustration of the transmission electron microscopy sample which is annealed in an oxidized/reduced atmosphere.

Supplementary Figure 8. The evolution of lattice structure at Fe-O layer in $Sr_4Fe_6O_{13-\delta}$ after being excited by the focused electron beam.

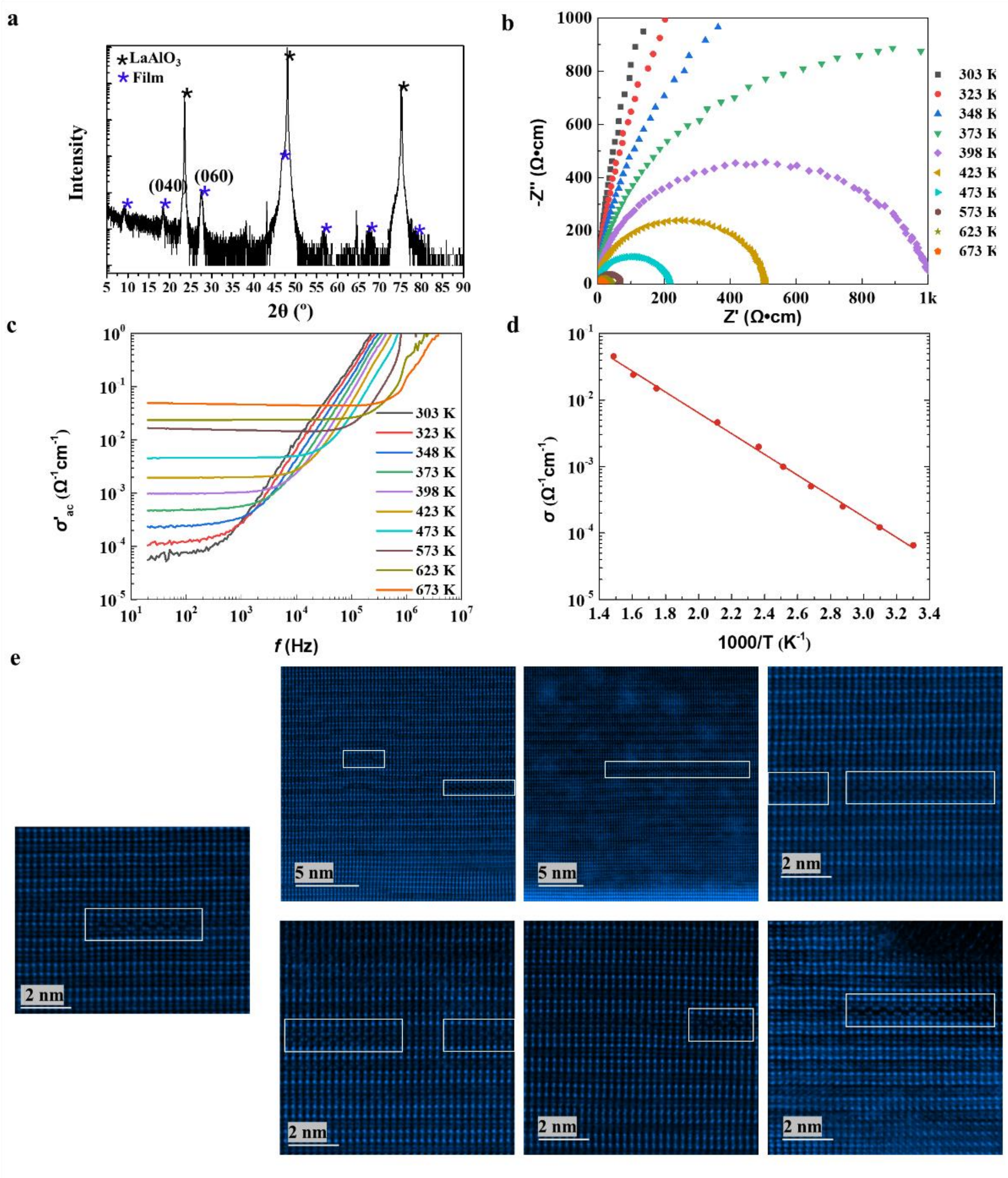

Supplementary Figure 1. X-ray Diffraction characterization and conductivity measurements of as-grown $Sr_4Fe_6O_{13-\delta}$ films (a) X-ray θ-2θ diffraction patterns of prepared epitaxial film on a $LaAlO_3$ substrate, where no additional peaks are observed. (b) The plot of representative impedance spectra measured at the different temperatures for the prepared films. (c) The plot of the real part of the lateral ionic conductivity versus frequency for the prepared film. (d) The lateral ionic conductivity versus inverse temperature. (e) HAADF-STEM images of different regions in prepared $Sr_4Fe_6O_{13-\delta}$ films. White rectangles indicate the tt-APBs.

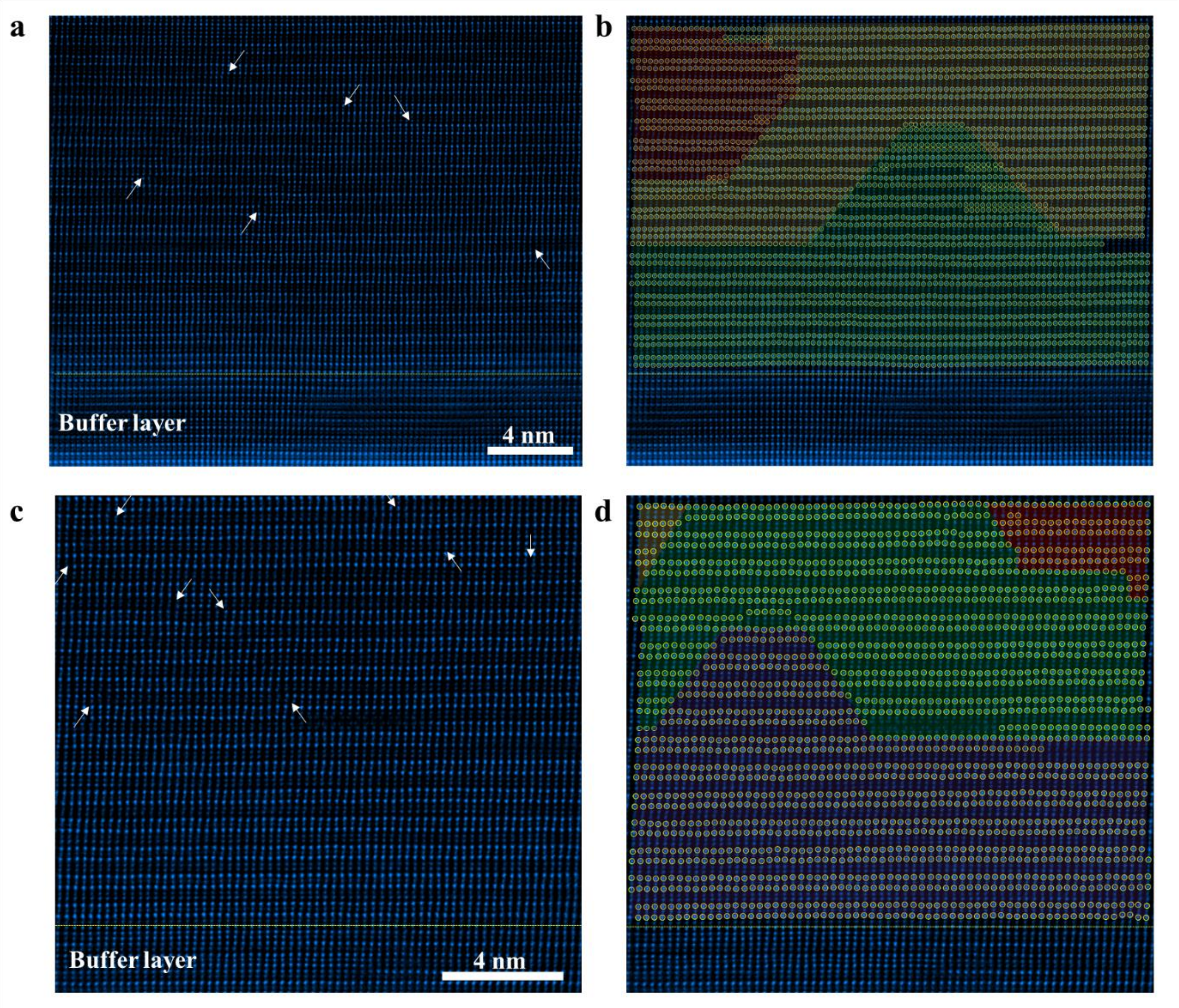

Supplementary Figure 2. Low-magnified cross-sectional HAADF STEM images of prepared $Sr_4Fe_6O_{13-\delta}$ films. Experimental HAADF STEM atomic image of prepared $Sr_4Fe_6O_{13-\delta}$ films in the region I (a) and region II (c) viewed along the cross-sectional direction. Regions with APBs-I are highlighted by white dotted arrows. It can be found that multiple APBs-I can be parallel or nearly symmetric to each other. Sr atom positions in the images of the (b) region I and the (d) region II are marked with yellow circles, to highlight the interfaces of APBs between adjacent domains with the atomic layer shifting against each other along out-of-plane direction.

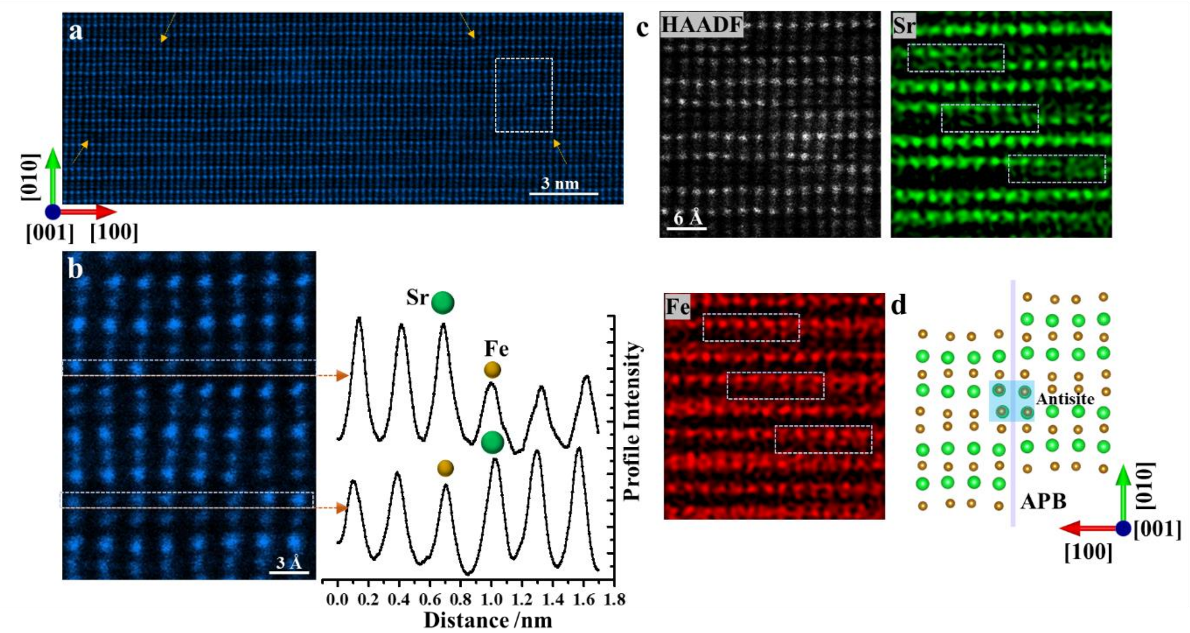

Supplementary Figure 3. Atomic-scale composition analysis of APBs in as-grown $Sr_4Fe_6O_{13-\delta}$ films. (a) HAADF STEM image of APBs. (b) Line profile of intensity at different atomic columns across the APBs. (c) atomic-scale element mapping of Fe and Sr across the APBs. (d) The schematic picture illustrates the atomic position occupied by Sr and Fe across the APBs.

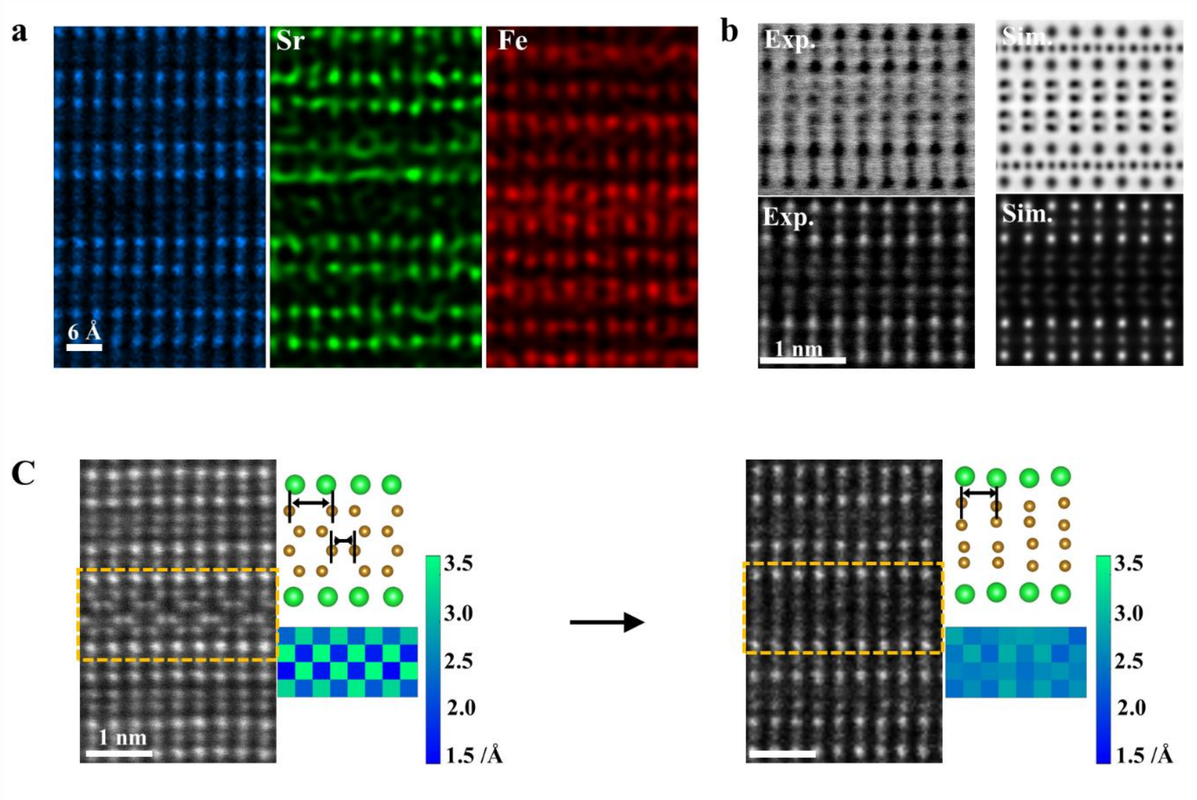

Supplementary Figure 4. Atomic-scale composition and structure analysis of four Fe-O layers at the topotactically transformable APBs-III, IV in as-prepared $Sr_4Fe_6O_{13-\delta}$ films. (a) Atomic-scale EDXS element mapping of four Fe-O layers after being oxidized. (b) Experimental and simulated ABF, HAADF STEM images of four Fe-O layers after being oxidized in as-prepared $Sr_4Fe_6O_{13-\delta}$ films. (c) HADDF STEM images of four Fe-O layers at the topotactically transformable APBs-III, IV before and after being oxidized in as-prepared $Sr_4Fe_6O_{13-\delta}$ films. Color maps on the left side refer to the distribution of lateral inter-atomic spacing (defined in the left schematic inset).

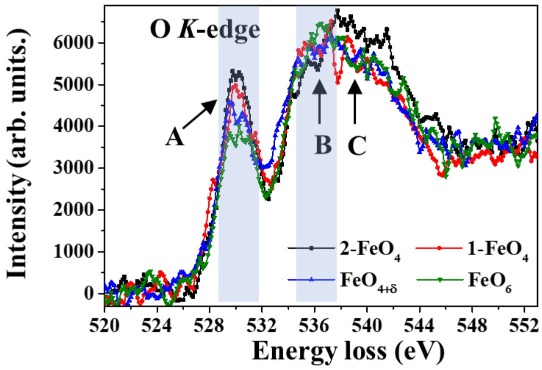

Layers	I_A/I_B
2-FeO_4	0.787
1-FeO_4	0.708
$FeO_{4+\delta}$	0.667
FeO_6	0.594

Supplementary Figure 5. The quantitative analysis of the ratios of integrated intensities of peak A and peak B.

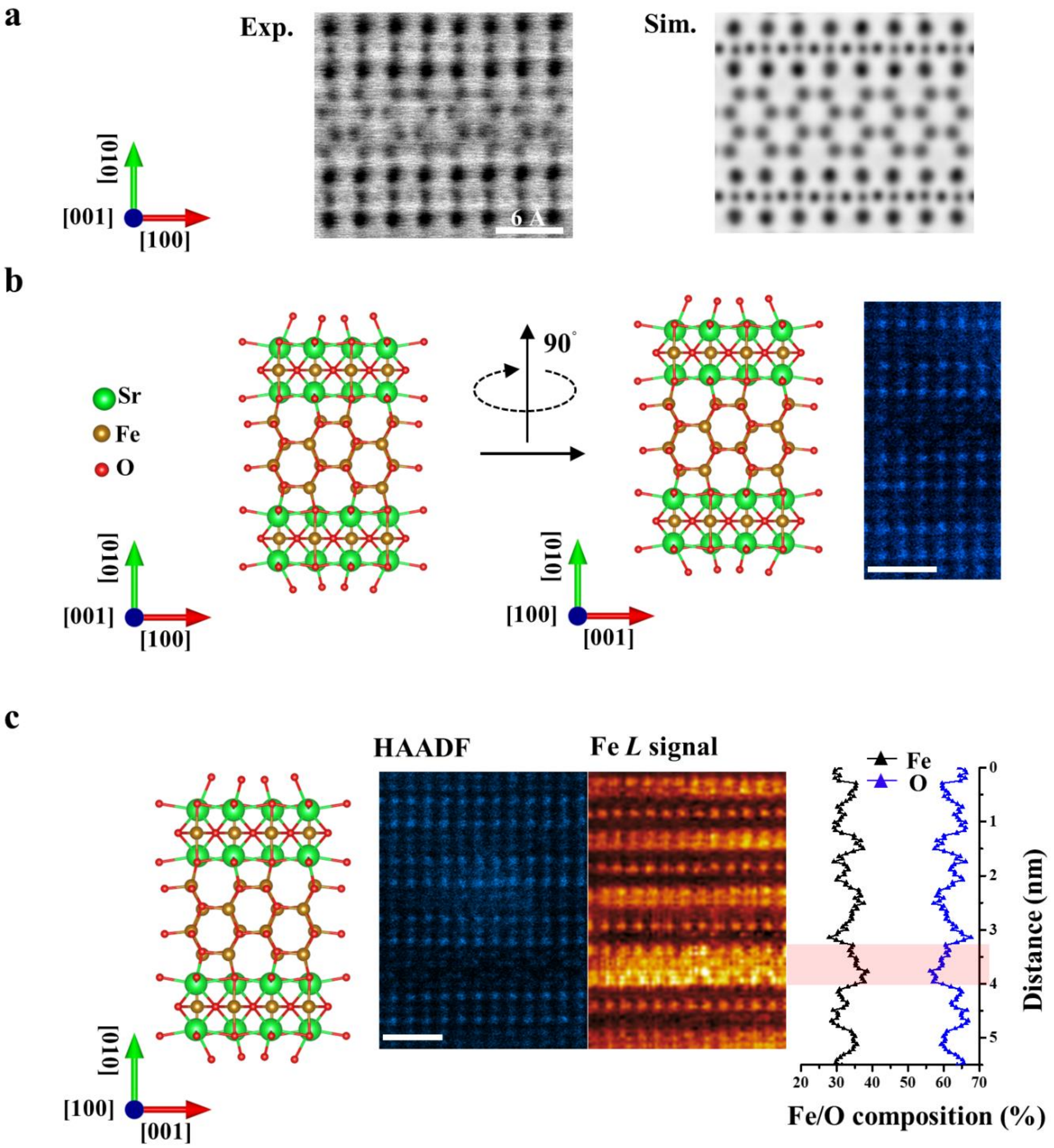

Supplementary Figure 6. Atomic-scale composition analysis of topotactically transformable APB-III. (a) Experimental and simulated ABF STEM images along the [001] zone axis. (b) HAADF STEM image of zigzag structure with four Fe-O layers along [100] direction. The scale bar is 1nm. (c) Fe element maps are extracted by using electron energy loss spectroscopy (EELS), and Fe/O relative content can be calculated as shown in the plot as the function of different layers of tt-APBs. The scale bar is 1 nm.

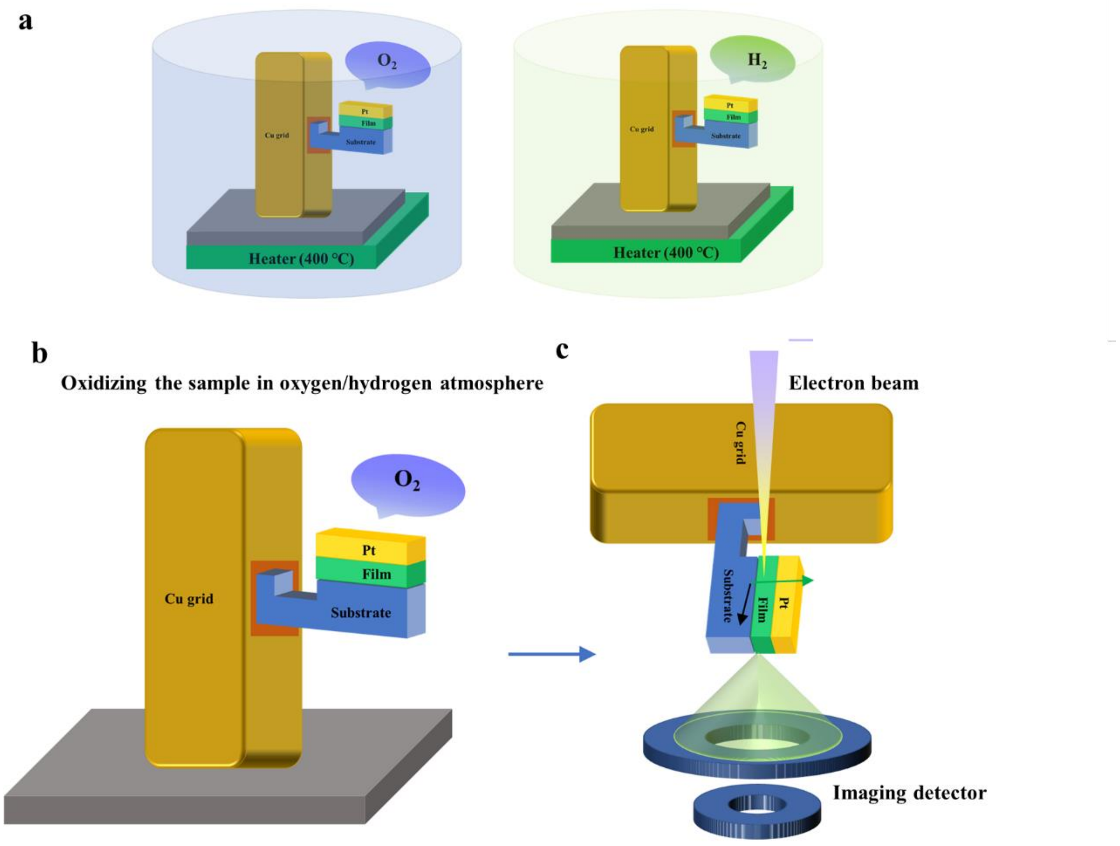

Supplementary Figure 7. The schematic illustration of the TEM sample annealed in a reduced/oxidized atmosphere. (a) Schematic model of the annealing process of reduction and re-oxidation for TEM samples. (b) A cross-sectional TEM sample is fixed at the Cu grid by using a focused ion beam, and then the TEM specimen can be annealed in an oxygen-enriched atmosphere. (c) Finally, the oxidized cross-sectional sample can be observed in the TEM instrument.

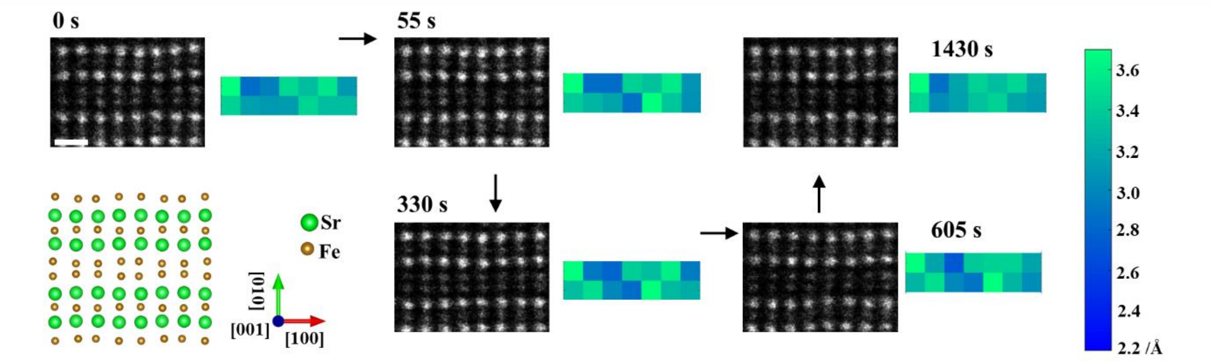

Supplementary Figure 8. The evolution of local lattice structure at Fe-O layer in $Sr_4Fe_6O_{13-\delta}$ after being excited by the electron beam. The experiment setup is the same as in Figure 4. The scale bar is 5 Å.
